# Treatment with TO901317, a synthetic liver X receptor agonist, reduces brain damage and attenuates neuroinflammation in experimental intracerebral hemorrhage

**DOI:** 10.1186/s12974-016-0524-8

**Published:** 2016-03-11

**Authors:** Chun-Hu Wu, Chien-Cheng Chen, Chai-You Lai, Tai-Ho Hung, Chao-Chang Lin, Min Chao, Szu-Fu Chen

**Affiliations:** Department of Physiology and Biophysics, National Defense Medical Center, Taipei, Taiwan, Republic of China; Graduate Institute of Life Sciences, National Defense Medical Center, Taipei, Taiwan, Republic of China; Department of Physical Medicine and Rehabilitation, Cheng Hsin General Hospital, Taipei, Taiwan, Republic of China; Department of Obstetrics and Gynecology, Chang Gung Memorial Hospital at Taipei and College of Medicine, Chang Gung University, Taipei, Taiwan, Republic of China; School of Medicine, National Defense Medical Center, Taipei, Taiwan, Republic of China

**Keywords:** Liver X receptors, Microglia, Inflammation, Intracerebral hemorrhage, Neuronal damage, TO901317

## Abstract

**Background:**

Intracerebral hemorrhage (ICH) induces a series of inflammatory processes that contribute to neuronal damage and neurological deterioration. Liver X receptors (LXRs) are nuclear receptors that negatively regulate transcriptional processes involved in inflammatory responses, but their role in the pathology following ICH remains unclear. The present study investigated the neuroprotective effects and anti-inflammatory actions of TO901317, a synthetic LXR agonist, in a model of collagenase-induced ICH and in microglial cultures.

**Methods:**

Mice subjected to collagenase-induced ICH injury were injected with either TO901317 (30 mg/kg) or vehicle 10 min after ICH and subsequently daily for 2 days. Behavioral studies, histology analysis, and assessments of hematoma volumes, brain water content, and blood-brain barrier (BBB) permeability were performed. The protein expression of LXR-α, LXR-β, ATP binding cassette transporter-1 (ABCA-1), and inflammatory molecules was analyzed. The anti-inflammatory mechanism of TO901317 was investigated in cultured microglia that were stimulated with either lipopolysaccharide (LPS) or thrombin.

**Results:**

ICH induced an increase in LXR-α protein levels in the hemorrhagic hemisphere at 6 h whereas LXR-β expression remained unaffected. Both LXR-α and LXR-β were expressed in neurons and microglia in the peri-ICH region and but rarely in astrocytes. TO901317 significantly attenuated functional deficits and brain damage up to 28 days post-ICH. TO901317 also reduced neuronal death, BBB disruption, and brain edema at day 4 post-ICH. These changes were associated with marked reductions in microglial activation, neutrophil infiltration, and expression levels of inflammatory mediators at 4 and 7 days. However, TO901317 had no effect on matrix metalloproteinase-9 activity. In BV2 microglial cultures, TO901317 attenuated LPS- and thrombin-stimulated nitric oxide production and reduced LPS-induced p38, JNK, MAPK, and nuclear factor-kappa B (NF-κB) signaling. Moreover, delaying administration of TO901317 to 3 h post-ICH reduced brain tissue damage and neuronal death.

**Conclusions:**

Our results suggest that enhancing LXR activation may provide a potential therapy for ICH by modulating the cytotoxic functions of microglia.

**Electronic supplementary material:**

The online version of this article (doi:10.1186/s12974-016-0524-8) contains supplementary material, which is available to authorized users.

## Background

Intracerebral hemorrhage (ICH) accounts for approximately 15 % of all strokes with high mortality and morbidity [1]. ICH induces brain damage due to initial tissue disruption from hematoma compression and subsequent development of excitotoxicity, oxidative damage, and inflammation [2, 3]. Of these, cerebral inflammation is considered to be a key pathological factor of ICH-induced brain damage and correlates with deterioration and poor outcomes in patients [4]. Suppression of inflammatory responses has also been reported to improve both histological and functional outcomes and to reduce brain edema in animal studies [5–8]. Inflammation after ICH involves infiltration of peripheral inflammatory cells, activation of microglia, and overproduction of inflammatory mediators, such as cytokines, chemokines, and matrix metalloproteases (MMPs) via nuclear factor-kappa B (NF-κB) signaling [9]. These inflammatory events may induce blood-brain barrier (BBB) disruption and brain edema, which ultimately lead to neuronal death and neurological deterioration [9]. Thus, defining the signals that control cerebral inflammatory responses has important implications for modulating disease processes following ICH.

Liver X receptors (LXRs) are nuclear receptors that regulate lipid metabolism at the transcriptional level [10]. Two members of the LXR superfamily have been identified, LXR-α and LXR-β, both of which are present in the nervous system and which have highly similar DNA or ligand-binding domains [10, 11]. LXRs can be activated by natural ligands, such as oxysterols [12] or by synthetic agonists [13]. The activation of LXRs regulates the expression of several genes that are involved in cholesterol metabolism [10]. In addition, LXRs negatively regulate transcriptional processes that are involved in inflammatory responses [10]. Previous in vitro studies have shown that LXR agonists attenuate inflammation by inhibiting NF-κB activity, and the expression of inflammatory mediators, such as inducible nitric oxide synthase (iNOS), cyclooxygenase-2 (COX-2), and pro-inflammatory cytokines and chemokines in microglia and astrocytes [14–16]. The anti-inflammatory activities of LXRs have also been observed in animal models of Alzheimer’s disease [17], Parkinson’s disease [18], spinal cord injury [19], and cerebral ischemia [20, 21]. LXR agonists also exert neuroprotection [20, 22, 23] and attenuate functional deficits [20, 22, 24] in experimental cerebral ischemia. However, no information is available concerning the possible therapeutic efficacy and anti-inflammatory activity of LXR activation after hemorrhagic stroke.

The aim of the present study was to determine the neuroprotective effect of LXR activation using a synthetic agonist, TO901317, in a mouse model of ICH. We also examined whether LXR activation attenuated ICH-induced microglial activation in both cell and animal models.

## Methods

### Animals

All animal protocols were carried out according to the Guide for the Care and Use of Laboratory Animals published by the US National Institutes of Health (NIH Publication No. 85-23, revised 1996), and were approved by the Animal Research Committee at Cheng Hsin General Hospital. Adult male C57BL/6J mice (age 8–10 weeks, weight 23–28 g) were kept at a constant temperature (21–25 °C) under a 12-h light/dark cycle with a humidity of approximately 45–50 %. Water and pellet chow were provided ad libitum.

### Experimental protocol

**Fig. 1:**
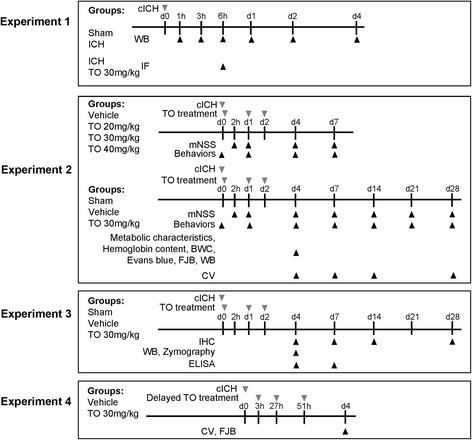
Experimental design and animal group classification. *cICH* collagenase-induced intracerebral hemorrhage, *IF* immunofluorescence staining, *TO* TO901317, *mNSS* modified neurological severity score, *BWC* brain water content, *FJB* Fluoro-Jade B staining, *WB* western blot, *CV* cresyl violet, *IHC* immunohistochemal staining, *ELISA* enzyme-linked immunosorbent assay

Mice were randomized into different treatment groups by using computer-generated random numbers. All outcome measurements and analyses described below were performed in a blinded manner. Four studies were conducted (Fig. 1). The first study examined the temporal profile and cellular localization of LXR expression after ICH. Assessment included western blots (*n* = 7/group) and double immunofluorescence labeling (*n* = 6/group). The second study evaluated the neuroprotective effect of the synthetic LXR agonist TO901317, which has been widely used in experimental studies and activates both LXR isoforms with similar potency [25]. TO901317 (30 mg/kg, Cayman Chemical, Ann Arbor, MI, USA) dissolved in either 30 % dimethyl sulfoxide (DMSO) (100 μL) or a corresponding volume of vehicle (30 % DMSO) was administered intraperitoneally (ip) 10 min after ICH and subsequently daily for 2 days (10 min, 24 h, and 48 h). Testing was as follows: (1) behavioral tests (*n* = 13/group); (2) metabolic characteristics and histology (*n* = 5–7/group); (3) hemoglobin assay, brain water content, and Evans Blue dye extravasation (*n* = 6–8/group); and (4) western blot analysis (*n* = 6–8/group). The dose and route of TO901317 were selected based on previous work on experimental cerebral ischemia [20, 21, 23] and on our pilot study, in which concentrations of 20, 30, and 40 mg/kg were tested. TO901317 at 30 and 40 mg/kg, but not at 20 mg/kg, reduced ICH-induced behavioral deficits with equivalent efficacy (Additional file 1: Figure S1). The three-dose regimen was chosen because inflammatory-related signals peak between 1 and 3 days after collagenase-induced ICH and decline thereafter [26, 27].

The third study investigated the anti-inflammatory effect of TO901317. Assessments included matrix metalloproteinase-9 (MMP-9) zymography and western blot analysis, enzyme-linked immunosorbent assay (ELISA), and immunohistochemistry (*n* = 5–7/group). The fourth study examined the delayed therapeutic potential of TO901317 for ICH. Either TO901317 or vehicle was administered ip at 3 h following ICH and subsequently daily for 2 days (3, 27, and 51 h), and protective effects were assessed using cresyl violet staining and Fluoro-Jade B (FJB) histology (*n* = 7/group).

### Intracerebral hemorrhage model

ICH model was performed as previously described [28]. Briefly, mice were placed into a stereotaxic frame after anesthesia with sodium pentobarbital (65 mg/kg, ip; Rhone Merieux, Harlow, UK). After retracting the scalp, a 30-gauge needle attached to a Hamilton syringe was implanted through a 1-mm-diameter burr hole into the right striatum (stereotaxic coordinates: 0.8 mm anterior and 2.5 mm lateral to bregma, 2.5 mm in depth). Bacterial collagenase (type VII-S; Sigma, St. Louis, MO, USA; 0.15 U in 0.5 μL of saline) was infused into the brain at a rate of 0.05 μL/min over 10 min with an infusion pump to induce intracerebral hemorrhage, and the needle was left in place for an additional 20 min to prevent reflux. After removal of the needle, the craniotomy was sealed with dental cement and the scalp was sutured closed. Mice were maintained at a 37.0 ± 0.5 °C body temperature using a heated pad throughout the surgery and recovery period. Sham-operated mice received an equal volume of normal saline in the same manner.

### Metabolic characteristics assessment

Mice were anesthetized with an overdose of sodium pentobarbital (200 mg/kg, ip), and right atrial puncture was performed to collect venous blood. The collected blood was centrifuged (3500 g for 5 min), and the serum was stored on ice until analysis. Serum blood urea nitrogen (BUN), creatinine (CRE), alanine aminotransferase (ALT), triglyceride (TG), and cholesterol (CHO) were measured by a chemistry autoanalyzer (Synchron Clinical System LX20; Beckman Coulter, Fullerton, CA, USA) to assess renal, liver, and cholesterol metabolisms.

### Behavioral testing

Behavioral recovery was assessed using modified neurological severity score (mNSS) test, rotarod test, and beam walking test. Animals were pre-trained for 3 days for both rotarod and beam walking tests.

#### Modified neurological severity score

The mNSS included motor, sensory, reflex, and balance tests [29]. A higher score represented a more severe injury. Neurological function was graded on a scale of 0–18 (normal score, 0; maximal deficit score, 18).

#### Rotarod test

An accelerating rotarod was used to measure motor function and balance in mice [30]. The rotarod speed was slowly increased from 6 to 42 rpm within 7 min, and the time for mice to fall off was recorded.

#### Beam walking test

The test was used to evaluate fine motor coordination and function by measuring the ability of the animals to traverse an elevated narrow beam as described previously [30].

For the rotarod and beam walking tests, three measurements per trial were recorded 1 h before ICH (baseline) and at 1, 4, 7, 14, 21, and 28 days after ICH.

### Hemoglobin assay

The hemoglobin contents of ICH brains were quantified using a spectrophotometric assay as previously described [28]. Mice were transcardially perfused and both ipsilateral and contralateral striatum regions were collected following ICH. Distilled water (300 μL) was added to each hemisphere, followed by homogenization for 30 s, sonication on ice for 1 min, and centrifugation at 13,000 rpm for 30 min. Drabkin reagent (80 μL; Sigma) was added to a 20-μL aliquot of supernatant (which contained the hemoglobin) and allowed to stand for 15 min at room temperature. Optical density was then measured at a wavelength of 545 nm to assess the concentration of cyanmethemoglobin. To generate a standard curve, blood was obtained by cardiac punctures in anesthetized control mice. Incremental volumes of this blood (0, 0.5, 1.0, 2.0, 4.0, and 8.0 μL) were then added to 300 μL of tissue lysate from a normal hemispheric sample.

### Brain water content

Brain water content was used as a measure of brain edema, which occurred because of BBB breakdown post-ICH. Following terminal anesthesia, mice were decapitated after ICH. The brains were immediately removed and divided into five parts, consisting of the ipsilateral and contralateral cortexes (CX), ipsilateral and contralateral basal ganglia (BG), and the cerebellum (which served as an internal control). Brain samples were immediately weighed on an electric analytical balance to obtain the wet weight and then dried at 100 °C for 24 h to obtain the dry weight. The water content of each sample was calculated using the following formula: [(wet weight − dry weight)/wet weight] × 100 %.

### Blood-brain barrier permeability

A 2 % solution of Evans Blue dye in normal saline (4 mL/kg of body weight) was injected into the tail vein and was allowed to circulate for 1 h. The mice were then transcardially perfused with phosphate-buffered saline (PBS) following terminal anesthesia, and samples from both hemispheres were homogenized in 1000 μL of trichloroacetic acid, sonicated, and centrifuged (15 min, 4500 rpm, 4 °C). The absorbance of each supernatant for Evans Blue dye was measured at 620 nm using a spectrophotometer. Evans Blue dye concentrations were calculated and expressed as micrograms per gram of brain tissue against a standard curve.

### Tissue processing and histology

Following terminal anesthesia, mice were transcardially perfused with PBS and then 4 % paraformaldehyde after ICH or sham surgery. Brains were removed, post-fixed in 4 % paraformaldehyde overnight, cryoprotected with 30 % sucrose, and then sectioned coronally (10 μm) over the entire region of injury.

### Hemorrhagic injury and hemispheric enlargement analysis

Injury volume, hemispheric atrophy, striatal atrophy, and hemispheric enlargement ratios were quantified using coronal sections stained with cresyl violet at 20 rostral-caudal levels that were spaced 200 μm apart. Sections were analyzed using ImageJ software version 1.48 (ImageJ, National Institutes of Health, Bethesda, MD, USA). Volume measurement was computed by a summation of the areas multiplied by the interslice distance (200 mm). Hemispheric or striatal atrophy was assessed using the following formula: ([Contralateral hemisphere or striatal volume − ipsilateral hemisphere or striatal volume]/contralateral hemisphere or striatal volume) × 100 %. Hemispheric enlargement was assessed using the following formula: ([ipsilateral hemisphere volume − contralateral hemisphere volume]/contralateral hemisphere volume) × 100 %. Analysis was performed by two experimenters who were blinded to all animal groups. Inter-rater reliability was within 10 %.

### Double immunofluorescence

To assess the cellular localization of LXR-α and LXR-β, double immunofluorescence labeling was performed by simultaneous incubation of either mouse anti-LXR-α (1:200; Abcam, Cambridge, MA, USA) or rabbit anti-LXR-β (1:50; Santa Cruz Biotechnology, Santa Cruz, CA, USA) with mouse anti-neuronal nuclei antigen (NeuN; a neuronal marker; 1:100; Millipore, Billerica, MA, USA), rabbit anti-microtubule-associated protein 2 (MAP2; a neuronal marker; 1:200; Millipore), rat anti-glial fibrillary acidic protein (GFAP; an astrocyte marker; 1:500; Invitrogen, Camarillo, CA, USA), rabbit anti-ionized calcium-binding adaptor molecule 1 (Iba-1; a microglia/macrophage marker; 1:400; Wako, Richmond, VA, USA), or rat anti-F4/80 (a microglia/macrophage marker; 1:400; Serotec, Düsseldorf, Germany) overnight at 4 °C. Sections were then washed, incubated with Alexa Fluor 488- or Alexa Fluor 594-conjugated secondary antibodies (1:400; Molecular Probes, Eugene, OR, USA) for 2 h, observed under a fluorescence microscope (Olympus BX-51; Olympus, Tokyo, Japan), and photographed.

### Immunohistochemistry

Immunohistochemical analyses were carried out as previously described [31]. After quenching of endogenous peroxidase activity and blocking of nonspecific binding, sections were allowed to react with the primary antibodies (rabbit anti-myeloperoxidase [MPO; a neutrophil marker; 1:1000; Dako, Carpinteria, CA, USA], rabbit anti-Iba-1 [a microglia/macrophage marker; 1:1000; Wako], or rat anti-CD45 [a marker for microglia and all blood-born leukocytes, including macrophages, monocytes, neutrophils, and T cells; 1:100; BD Biosciences Pharmigen, San Jose, CA, USA]) at 4 °C overnight. Further colorimetric detection was processed according to the instructions of a Vectastain Elite ABC Kit (Vector Laboratories, Burlingame, CA, USA) with the use of diaminobenzidine as a peroxidase substrate. The specificity of the staining reaction was assessed in several control procedures, including omission of the primary antibody and substitution of the primary antibody with non-immune rabbit serum.

### Fluoro-Jade B histochemistry

FJB is a polyanionic fluorescein derivative that binds with high sensitivity and specificity to degenerating neurons. Briefly, sections were rehydrated in graded ethanol solutions (75, 50, and 25 %, 5 min each) and distilled water, incubated in 0.06 % KMnO_4_ for 10 min, rinsed in distilled water for 2 min, incubated in a 0.0004 % solution of FJB (Chemicon, Temecula, CA, USA) for 30 min, and observed under a fluorescence microscope (Olympus) at 450–490 nm.

### Quantification of FJB, Iba-1, MPO, and CD45 staining

For each animal, FJB, Iba-1, MPO, and CD45 staining or double immunofluoresence for cellular markers and LXRs were quantified on three consecutive sections from the hemorrhagic core at the level of 0.24 mm from the bregma. The number of positive cells was counted in an area of 920 × 860 μm^2^ in 10–12 non-overlapping fields immediately adjacent to the hematoma using a magnification of ×200 as previously described [28, 32]. Iba-1-positive resting microglia/macrophages were defined as resting if they contained relatively small cell bodies (<7.5 μm in diameter) with long slender processes [33]. Microglia were defined as activated when a cell body increased in size compared to resting microglia with short, thick processes and intense immunointensity. Activated microglia/macrophages were defined based on a combination of morphological criteria and a cell body diameter cutoff of 7.5 μm. The total number of FJB-, Iba-1-, and MPO-positive cells was expressed as the mean number per field of view. Quantification of LXRs in neurons or microglia was expressed as (LXR-stained neurons or microglia/neurons or microglia) × 100 %. Analysis was performed by two experimenters who were blinded to all animal groups. Inter-rater reliability was within 10 %.

### Western blot

Samples were collected and western blot was performed as previously described [28]. A 2-mm-thick coronal section from the ipsilateral hemisphere was collected following ICH or sham surgery. Equal amounts of protein (35 to 50 μg protein in 20 μL for tissue samples and 20 μg protein in 20 μL for cell lysates) were separated by sodium dodecyl sulfate-polyacrylamide gels, transferred to Immobilon-P membranes (Millipore), blocked using 5 % milk in PBS containing 0.1 % Tween-20, and probed overnight at 4 °C with primary antibodies including mouse anti-LXR-α (1:1000; Abcam), rabbit anti-LXR-β (1:1000; Santa Cruz), mouse anti-ATP binding cassette transporter-1 (ABCA-1; 1:1000; Abcam), mouse anti-iNOS (1:200; Sigma), rabbit anti-COX-2 (1:1000; Cayman), rabbit anti-NF-κB p65 (1:1000; Santa Cruz), rabbit phospho-p38 (1:1000; Cell Signaling, Danvers, MA, USA), rabbit total p38 (1:2000; Cell Signaling), rabbit phospho-Jun amino-terminal kinases (JNK; Thr183/Tyr185, 1:1000; Cell Signaling), rabbit total JNK (1:2000; Cell Signaling), rabbit phospho-extracellular signal-regulated kinases p44/42 (Erk p44/42; Thr202/Tyr204, 1:1000; Cell Signaling), and rabbit total Erk (1:2000; Cell Signaling). The membranes were then incubated with horseradish peroxidase-linked anti-rabbit or anti-mouse secondary antibodies (Santa Cruz Biotechnology; 1:1000) for 1 h at 4 °C. Protein band intensities were quantified using ImageJ software, and the relative intensity of protein signals was normalized to the corresponding β-actin intensity.

### Gelatin zymography

Zymography was performed as previously described [28]. Briefly, protein samples were equally loaded and separated by a 10 % Tris-glycine gel with 0.1 % gelatin as a substrate. After separation, the gel was washed in distilled water twice for 30 min, re-natured for 1 h with 2.5 % Triton X-100 buffer at room temperature, and incubated for 48 h with developing buffer (0.05 M Tris-HCl, pH 7.5; 0.2 mol/L NaCl; 5 mmol/L CaCl_2_; 0.05 % Brij-35; and 0.2 mmol/L NaN_3_) at 37 °C. Following this, the gel was stained with 0.05 % Coomassie R-250 dye (Sigma) for 30 min and appropriately de-stained. Gelatinolytic activity (MMP-9, 92 kDa) was determined as by the appearance of clear bands at the appropriate molecular weights.

### Enzyme-linked immunosorbent assay and nitrite assay

Protein samples were collected as in western blotting following ICH or sham surgery. Monocyte chemoattractant protein-1 (MCP-1), macrophage inflammatory protein-2 (MIP-2), interleukin (IL)-6, and interleukin-1β (IL-1β) were measured in brain homogenates or cell lysates using a commercially available ELISA kit (R&D Systems, Minneapolis, MN, USA). All samples and standards were assayed in duplicate according to the manufacturer’s instructions. Nitric oxide (NO) production was assessed by measuring the nitrite levels of the culture supernatants with Griess reagent (Sigma).

### Microglial culture

The mouse microglial BV2 cell line was cultured in Dulbecco’s modified Eagle’s media (DMEM; Gibco/BRL, Bethesda, MD, USA) supplemented with 10 % heat-inactivated fetal bovine serum (FBS; Gibco/BRL), 100 U/mL penicillin and 100 μg/mL streptomycin in a humidified atmosphere of 5 % CO_2_ at 37 °C. BV2 microglia were stimulated with either 0.1 μg/mL lipopolysaccharide (LPS) or 10 U/mL thrombin in the absence or presence of varying concentrations of TO901317 for 24 h. The experiments were repeated four times with different batches of cultures.

### Statistical analyses

All data are presented as the mean and standard error of the mean (mean ± SEM). One-way or two-way analysis of variance (ANOVA) followed by post hoc Bonferroni evaluation was used for multiple groups to determine significant differences. Student’s *t* test was used to test the differences between two groups. Statistical significance was set at *P* < 0.05.

## Results

### Increased LXR-α protein expression in mice after ICH

**Fig. 2:**
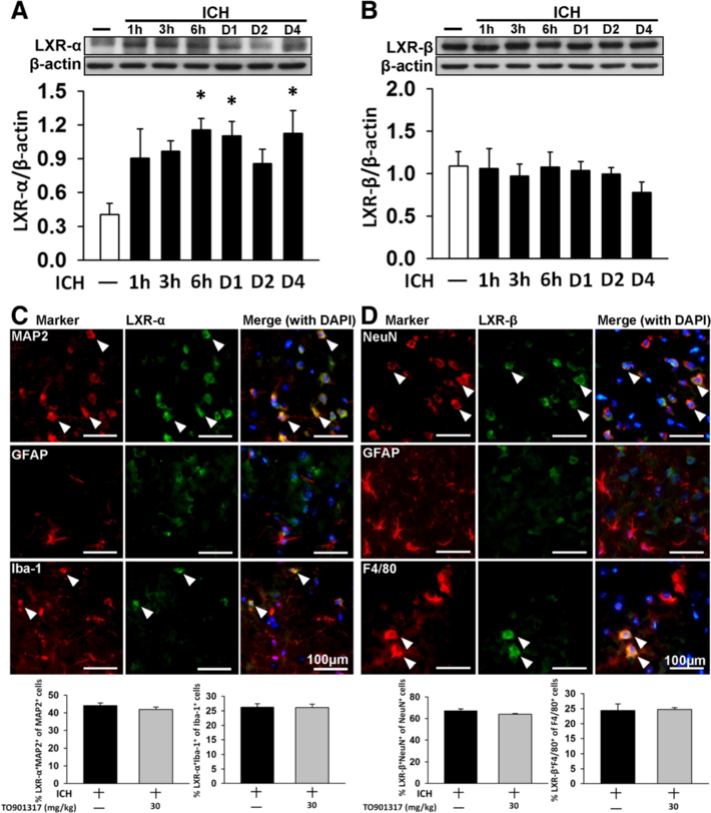
Expression and cellular distribution of LXR-α and LXR-β receptor isoforms in mouse brains subjected to ICH. Representative immunoblots and quantitative data of **a** the LXR-α and **b** LXR-β protein levels in the ipsilateral (hemorrhagic) hemispheres from ICH or sham-operated mice after collagenase injection. Bar graphs of densitometric analysis of bands show a significant increase in LXR-α protein levels in the ipsilateral hemispheres of ICH mice at 6 h, 1 day, and 4 days post-ICH, compared with the sham-operated mice. LXR-β expression remained unaffected after ICH. Values are mean ± SEM; ^*^ *P* < 0.05 versus sham group (*n* = 7 mice/group, one-way ANOVA). **c** LXR-α and **d** LXR-β in the peri-hematomal area observed by immunofluorescence labeling. LXRs are shown in *green*, and immunolabeling of MAP 2 or NeuN (neurons), Iba-1 or F4/80 (microglia), or GFAP (astrocytes) is shown in *red*. Yellow labeling (*white arrows*) indicates co-localization. Both LXR-α and LXR-β were mainly expressed in neurons and microglia and rarely in astrocytes. Sections were stained with DAPI (*blue*) to show all nuclei. The scale bar is 100 μm. Values are mean ± SEM (*n* = 6 mice/group, Student’s *t* test)

We first examined the temporal profiles and cellular localizations of LXRs following ICH in mice. ICH induced an increase in LXR-α protein levels in the hemorrhagic hemisphere at 6 h (284 % of the sham level; *P* = 0.019), 1 day (272 %; *P* = 0.038) and 4 days (277 %; *P* = 0.029), whereas LXR-β expression remained mostly unaffected (Fig. 2a, b). Because LXR-α expression peaked at 6 h after collagenase-induced ICH, we investigated the cellular localizations of LXRs at this time point. Dual-label immunofluorescence demonstrated that both LXR-α and LXR-β were mainly expressed in neurons and microglia in the peri-ICH region and rarely in astrocytes (Fig. 2c, d). Quantification results showed that in the vehicle-treated ICH group, 44.1 ± 1.5 % of neurons were positive for LXR-α and 67.1 ± 1.9 % of neurons were positive for LXR-β. For microglia/macrophages, 26.2 ± 1.2 % were positive for LXR-α and 24.4 ± 2.2 % were positive for LXR-β. Similarly, in the TO901317-treated group, 42.0 ± 1.2 % of neurons were positive for LXR-α and 64.1 ± 0.7 % of neurons were positive for LXR-β. For microglia/macrophages, 26.1 ± 1.2 % were positive for LXR-α and 24.8 ± 0.6 % were positive for LXR-β. There was no difference between the vehicle and TO901317-treated groups for the ratios of LXR-positive neurons or microglia.

### TO901317 improved long-term neurobehavioral function but did not alter hemorrhage size in mice after ICH

**Fig. 3:**
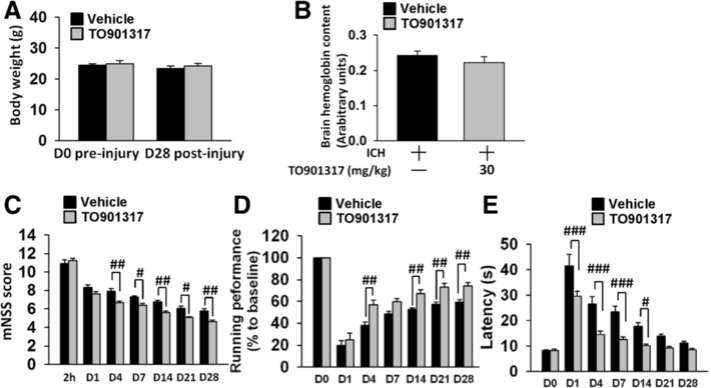
TO901317 improved neurobehavioral function but did not alter body weight or hemorrhage size after ICH. **a** There were no significant differences in body weight between the control (vehicle-treated) and 30 mg/kg TO901317-treated groups before or at 28 days after ICH. **b** Hemoglobin levels in vehicle-treated and TO901317-treated mice were not significantly different at 4 days post-ICH. Treatment with 30 mg/kg TO901317 (compared with vehicle) significantly **c** reduced the modified neurological severity score (mNSS) from 4 to 28 days (all *P* < 0.05), **d** improved the rotarod performance at 4, 14, 21, and 28 days (all *P* < 0.01), and **e** reduced the beam walk traversing time from 1 to 14 days (all *P* < 0.05) post-ICH. Values are mean ± SEM; ^#^ *P* < 0.05, ^##^ *P* < 0.01, and ^###^ *P* < 0.001 versus vehicle group (*n* = 13 mice/group, Student’s *t* test for body weight; *n* = 8 mice/group, Student’s *t* test for hemoglobin assay; *n* = 13 mice/group, repeated measures two-way ANOVA for behavioral tests)

To investigate the protective efficacy of LXR activation in ICH, TO901317 was employed to activate LXR following ICH. We first assessed the safety of TO901317 in mice on the 4th day post-ICH, at which point one dose of either vehicle or TO901317 was administered daily for 3 days. Treatment with TO901317 did not alter plasma concentrations of BUN and CRE, which are indicators of renal function, ALT, an indicator of hepatic function, or TG and CHO, which are indicators of cholesterol metabolism (Table 1). Similarly, no significant between-group differences were found in body weight change at 28 days (Fig. 3a) or in brain hemoglobin content, an indicator of hemorrhage size, at 4 days (Fig. 3b). We then assessed the protective effects of TO901317 on behavioral recovery following ICH using three different behavioral tests. The extent of global neurological deficit was evaluated by mNSS. At 2 h after injury, there was no difference in mNSS between vehicle-treated and TO901317-treated groups, indicating that injury severity was initially similar regardless of treatment (Fig. 3c). Significant improvement in neurological function was observed from 4 to 28 days in the TO901317 group compared with the vehicle group (all *P* < 0.05; Fig. 3c). Rotarod and beam walking tests were employed to evaluate motor and coordination functions. The TO901317 group had better rotarod performance compared to that of the vehicle group at 4, 14, 21, and 28 days (all *P* < 0.01; Fig. 3d). Likewise, beam walk latencies were shorter in the TO901317 group from 1 to 14 days (all *P* < 0.05; Fig. 3e).

**Table 1:** Metabolic characteristics of the mice treated with vehicle and TO901317 30 mg/kg

		ICH day 4	
	Vehicle	TO901317 30 mg/kg	Reference range
BUN (mg/dL)	20.60 ± 2.45	18.48 ± 3.92	8–33
CRE (mg/dL)	0.06 ± 0.02	0.05 ± 0.02	0.2–0.9
ALT (mg/dL)	25.00 ± 4.53	29.20 ± 4.47	17–77
TG (mg/dL)	78.00 ± 18.02	62.20 ± 28.79	60–160
CHO (mg/dL)	74.60 ± 12.46	73.40 ± 19.57	90–170

Values are expressed as means ± SEM. *n* = 5 mice/group [31, 57]

*ICH* intracerebral hemorrhage, *BUN* blood urea nitrogen, *CRE* creatinine, *ALT* alanine aminotransferase, *TG* triglyceride, *CHO* cholesterol

### TO901317 ameliorated brain tissue loss and neuronal damage in mice after ICH

**Fig. 4:**
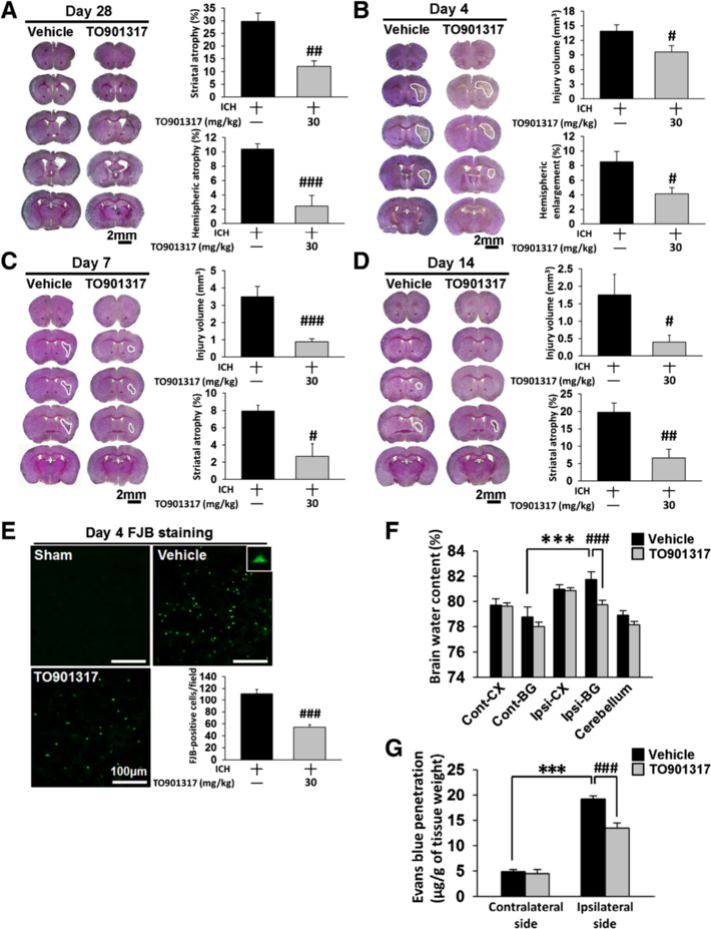
TO901317 attenuated brain tissue and neuronal damage reduced brain edema and BBB disruption after ICH. Representative cresyl violet-stained brain sections of vehicle-treated and 30 mg/kg TO901317-treated mice at **a** 28, **b** 4, **c** 7, and **d** 14 days post-ICH. Analysis of lesion volumes demonstrates that 30 mg/kg TO901317 significantly reduced hemispheric and striatal atrophy at 28 days and significantly reduced hemorrhagic injury volume and hemispheric enlargement at 4 days. TO901317 also attenuated hemorrhagic injury volume and striatal atrophy at 7 and 14 days. The scale bar is 2 mm. **e** Representative FJB-stained sections of a sham-injured, a vehicle-treated, and a 30 mg/kg TO901317-treated mouse at 4 days post-ICH. The *inset* is a representative FJB-positive cell at higher magnification. Quantification analysis shows that TO901317 significantly reduced the number of degenerating neurons at 4 days post-ICH. The scale bar is 100 μm. **f** Brain water content in the ipsilateral basal ganglion of vehicle-treated mice was significantly higher than in the contralateral basal ganglion at 4 days post-ICH. In the ipsilateral basal ganglion, brain water content of 30 mg/kg TO901317-treated mice was significantly lower than in vehicle-treated mice. *Cont-BG* contralateral basal ganglia, *Cont-CX* contralateral cortex, *Ipsi-BG* ipsilateral basal ganglia, *Ipsi-CX* ipsilateral cortex. **g** TO901317 (30 mg/kg) significantly decreased leakage of Evans Blue dye into the brain in the ipsilateral hemisphere compared with the vehicle-treated mice. Values are mean ± SEM; ^***^ *P* < 0.001 versus contralateral hemisphere, ^#^ *P* < 0.05, ^##^ *P* < 0.01, and ^###^ *P* < 0.001 versus vehicle group (*n* = 6–8 mice/group, Student’s *t* test)

To determine whether the above changes in behavioral function reflected in a reduction of brain tissue damage and neuronal death, histological outcomes were evaluated. ICH induced pronounced loss of tissue in the hemorrhagic hemisphere and striatum at day 28 post-ICH (Fig. 4a). However, TO901317 significantly reduced the degree of hemispheric atrophy (2.5 ± 1.5 % versus 10.4 ± 0.7 %, *P* < 0.001; Fig. 4a) and striatal atrophy compared to vehicle (11.6 ± 2.6 % versus 31.9 ± 4.0 %, *P* = 0.0029) at 28 days. We further evaluated whether TO901317 attenuated brain tissue damage and neuronal injury during the earlier stage of ICH. Consistent with the protective effect at 28 days, TO901317 treatment significantly reduced hemorrhagic injury volume to 69 % of the vehicle group (9.5 ± 1.3 mm^3^ versus 13.9 ± 1.3 mm^3^, *P* = 0.047; Fig. 4b) at 4 days. Hemispheric enlargement, an indicator of brain edema, was also significantly smaller in TO901317-treated mice (4.1 ± 0.8 %) than in vehicle-treated mice (8.5 ± 1.4 %, *P* = 0.02; Fig. 4b) at 4 days. Moreover, TO901317 significantly attenuated hemorrhagic injury volume at both 7 (0.9 ± 0.2 mm^3^ versus 3.5 ± 0.6 mm^3^, *P* < 0.001; Fig. 4c) and 14 days (0.4 ± 0.2 mm^3^ versus 1.8 ± 0.6 mm^3^, *P* = 0.0426; Fig. 4d). At 7 and 14 days after ICH, tissue loss was primary in the ipsilateral striatum, and the total hemispheric atrophy was more evident at 28 days. TO901317 significantly reduced the degree of striatal atrophy at 7 (2.7 ± 1.5 % versus 7.9 ± 0.6 %, *P* = 0.0133; Fig. 4c) and 14 days (6.7 ± 2.5 % versus 19.8 ± 2.6 %, *P* = 0.0066; Fig. 4d). In parallel with the effect of brain tissue protection, TO901317 significantly attenuated the number of FJB-positive degenerative neurons around the hematoma when compared to vehicle administration at 4 days post-ICH (54.3 ± 4.3 versus 111.3 ± 7.6 cells/field, *P* < 0.001; Fig. 4e).

### TO901317 attenuated brain edema and BBB permeability in mice after ICH

We next explored whether TO901317 influenced ICH-induced brain edema and BBB breakdown as both are consequences of inflammatory responses following hemorrhagic stroke [34]. Brain water content was significantly increased in the ipsilateral hemisphere in the vehicle group compared with the contralateral counterpart (82.0 ± 0.5 % versus 78.4 ± 0.6 %; *P* < 0.001) at 4 days post-ICH, which was significantly decreased with TO901317 treatment (79.2 ± 0.3 % versus 82.0 ± 0.5 %, *P* < 0.001; Fig. 4f). To further examine the effects of TO901317 on BBB permeability, we used Evans Blue dye as a marker for albumin extravasation. ICH resulted in significantly increased Evans Blue extravasation in the hemorrhagic hemisphere compared to the contralateral side at 4 days post-ICH (19.2 ± 0.7 versus 4.9 ± 0.4 μg/g, *P* < 0.001; Fig. 4g). However, TO901317 administration resulted in attenuated levels of extracted Evans Blue compared to vehicle-treated mice at 4 days (13.5 ± 1.0 versus 19.2 ± 0.7 μg/g, *P* < 0.001; Fig. 4g).

### TO901317 activated the LXR target gene ABCA-1 in mice after ICH

**Fig. 5:**
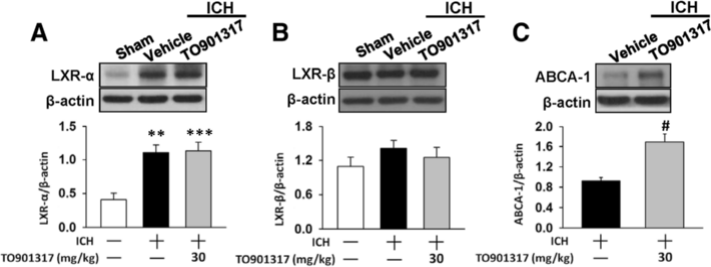
TO901317 did not affect LXR expression but increased the LXR target gene, ABCA-1 protein expression post-ICH. Representative immunoblots of **a** LXR-α, **b** LXR-β, and **c** ABCA-1 proteins in the ipsilateral hemisphere of sham-injured, vehicle-treated, and 30 mg/kg TO901317-treated mice at 4 days post-ICH. Bar graphs of densitometric analysis of bands show that TO901317 did not affect either LXR-α or LXR-β protein expression but increased the LXR target gene, ABCA-1 protein expression. Values are mean ± SEM; ^**^ *P* < 0.01, and ^***^ *P* < 0.001 versus sham group, ^#^ *P* < 0.05 versus vehicle group (*n* = 5–7 mice/group, one-way ANOVA for LXRs and Student’s *t* test for ABCA-1 western blot).

To determine whether TO901317 acted through LXR, we examined the expression levels of LXRs and their target gene ABCA-1 in ICH following the administration of TO901317. ICH induced a significant increase in LXR-α protein expression compared to the sham control at day 4, whereas LXR-β expression remained unchanged (Fig. 5a, b). Both LXR-α and LXR-β protein levels were unaltered in the ICH mice after TO901317 treatment compared with vehicle treatment. However, the protein level of ABCA-1, a representative LXR-regulated gene, was significantly elevated following TO901317 administration at day 4 post-ICH (*P* = 0.019; Fig. 5c). These results suggest that brain LXRs were activated in a ligand-dependent manner after ICH.

### TO901317 attenuated neutrophil infiltration, microglial activation, and macrophage infiltration but did not affect MMP-9 activity in mice after ICH

**Fig. 6:**
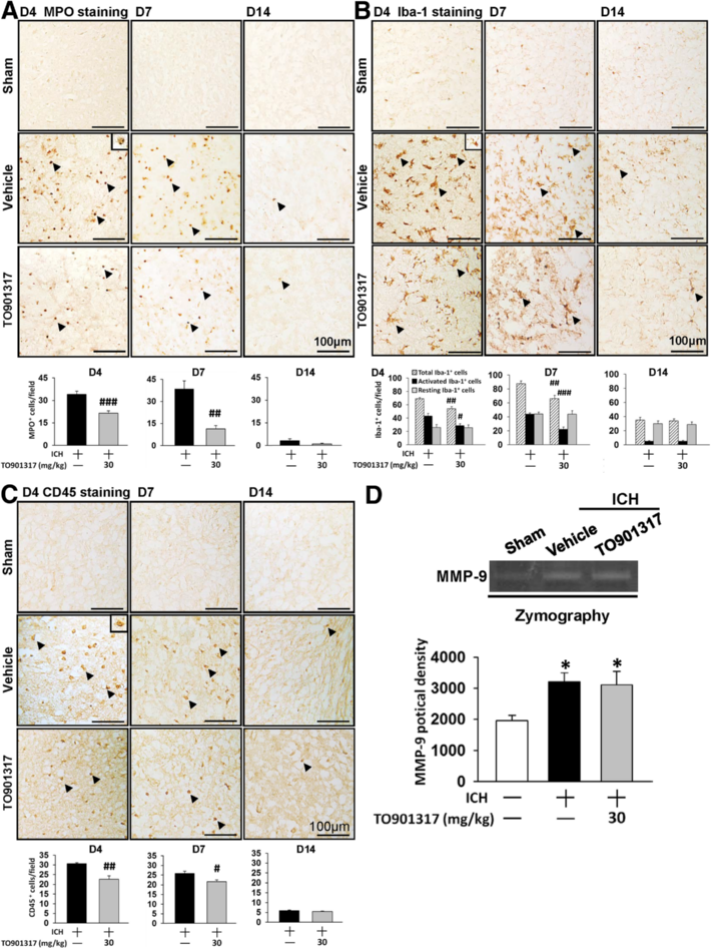
TO901317 reduced neutrophil infiltration and microglial activation but had no effect on MMP-9 activity. Representative **a** MPO-stained, **b** Iba-1-stained, and **c** CD45-stained brain sections from sham-injured, vehicle-treated, and 30 mg/kg TO901317-treated mice at 4, 7 and 14 days post-ICH. Cell count analysis shows that TO901317-treated mice had significantly fewer MPO-positive, total Iba-1-positive, activated Iba-1-positive, and CD45-positive cells than vehicle-treated mice in the peri-hematoma area at 4 and 7 days post-ICH. The number of MPO-positive, Iba-1-positive, and CD45 positive cells is expressed as the mean number per field of view (0.8 mm^2^). The scale bar is 100 μm. **d** Representative zymography of MMP-9 activity from a sham-injured control, a vehicle-treated, and a 30 mg/kg TO901317-treated mouse at 1 day post-ICH. There was no difference in MMP-9 activity between vehicle-treated and TO901317-treated mice. Values are mean ± SEM; ^*^ *P* < 0.05 versus sham group, ^#^ *P* < 0.05, ^##^ *P* < 0.01, and ^###^ *P* < 0.001 versus vehicle group (*n* = 6–7 mice/group, Student’s *t* test for MPO, Iba-1, and CD45 immunohistochemistry; *n* = 6 mice/group, one-way ANOVA for MMP-9 zymography)

We next evaluated degrees of neutrophil infiltration and microglial activation to determine the anti-inflammatory activity of TO901317. ICH resulted in an accumulation of neutrophils in the peri-hematoma area at 4 days (34.0 ± 2.1 cells/field) and 7 days (38.5 ± 5.6 cells/field), and TO901317 treatment significantly reduced this response (21.4 ± 1.6 cells/field at 4 days, *P* < 0.001; 11.4 ± 2.2 cells/field at 7 days, *P* = 0.0011; Fig. 6a). The infiltrated neutrophils were nearly absent in the peri-hematoma area at 14 days. The number of total microglia/macrophages (Iba-1-positive) cells in the hemorrhagic brain also significantly increased compared with the sham group (38.8 ± 2.2 cells/field) in the peri-hematoma area at both 4 days (69.2 ± 1.6 cells/field) and 7 days (87.9 ± 3.7 cells/field; Fig. 6b). On the other hand, animals who received TO901317 treatment showed significant reductions in the total number of Iba-1-positive cells at both 4 days (54.1 ± 3.5 cells/field, *P* = 0.002) and 7 days (66.1 ± 4.9 cells/field, *P* = 0.005) following ICH. There was no difference between the vehicle and TO901317-treated groups at 14 days (35.1 ± 3.8 versus 33.7 ± 3.1 cells/field, *P* = 0.785). There was also a large number of activated microglia/macrophages in the peri-hematoma area at 4 and 7 days after ICH, which was scant at 14 days. TO901317 significantly reduced the number of activated Iba-1-positive cells at 4 days (28.6 ± 3.1 cells/field versus 43.1 ± 4.0 cells/field, *P* = 0.013, Fig. 6b) and 7 days (22.2 ± 3.2 cells/field versus 43.8 ± 1.9 cells/field, *P* < 0.001). However, TO901317 treatment did not affect the number of resting microglia at both 4 and 7 days. We then used CD45 staining, an antigen associated with microglia and all leukocytes, including macrophages, monocytes, neutrophils, and T cells, to identify inflammatory cells. The expression of CD45 is low in resting microglia and increases during microglial activation, whereas CD45 expression is high in blood-born monocytes [35, 36]. Similarly, TO901317 treatment significantly reduced the number of CD45-positive cells in the peri-hematoma area at 4 days (22.6 ± 1.7 cells/field versus 30.6 ± 0.5 cells/field, *P* = 0.0043; Fig. 6c) and 7 days (21.5 ± 1.0 cells/field versus 25.7 ± 1.3 cells/field, *P* = 0.0256) compared with the control group. Both activated Iba-1- and CD45-positive cells were scant at 14 days following ICH. We further analyzed MMP-9 activity, which mediates BBB breakdown by degrading the basal lamina and contributes to the pathophysiology of ICH. MMP-9 activity was significantly increased at 4 days post-ICH, but there was no difference in MMP-9 activity between vehicle-treated and TO901317-treated mice (Fig. 6d).

### TO901317 reduced expression of inflammatory mediators in mice after ICH

**Fig. 7:**
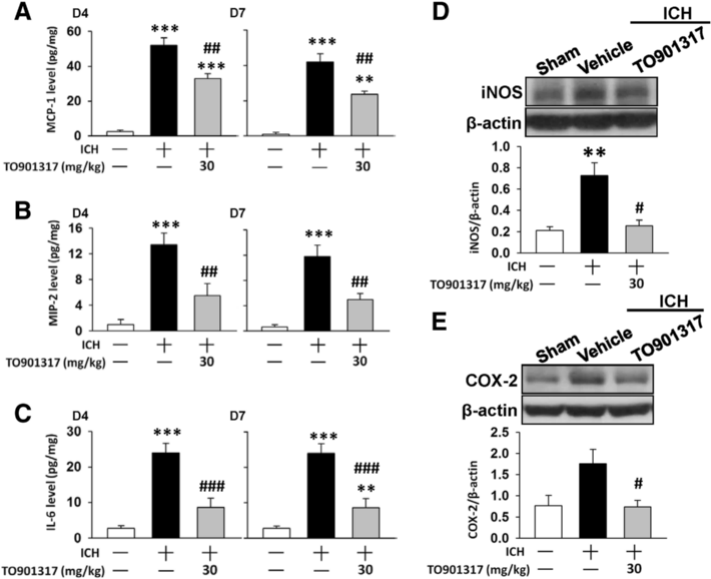
TO901317 reduced expression of inflammatory mediators in mice after ICH. Bar graphs of **a** MCP-1, **b** MIP-2, and **c** IL-6 protein concentrations, as assessed by ELISA in ipsilateral hemispheres of sham control, vehicle-treated, and 30 mg/kg TO901317-treated mice at 4 and 7 days post-ICH. TO901317-treated mice exhibited significantly reduced MCP-1, MIP-2, and IL-6 protein levels compared with vehicle-treated mice at 4 and 7 days. Representative immunoblots of **d** iNOS and **e** COX-2 proteins in ipsilateral hemispheres of sham control, vehicle-treated, and 30 mg/kg TO901317-treated mice at 4 days post-ICH. Bar graphs of densitometric analysis of bands show a significant decrease of iNOS and COX-2 protein levels in ipsilateral hemispheres of TO901317-treated mice, compared with vehicle-treated mice. Values are presented as means ± SEM; ^**^ *P* < 0.01 and ***P < 0.001 versus sham control; ^#^ *P* < 0.05, ^##^ *P* < 0.01, ^###^ *P* < 0.001 versus vehicle group (*n* = 6–7 mice/group, one-way ANOVA)

We then assessed whether TO901317 influenced the expression of inflammatory mediators following ICH. ICH induced an increase in MCP-1, MIP-2, and interleukin-6 (IL-6) protein expression in hemorrhagic hemispheres at 4 and 7 days, but treatment with TO901317 significantly reduced the MCP-1, MIP-2, and IL-6 levels at both 4 days (MCP-1: 33.1 ± 2.7 versus 52.1 ± 4.2 pg/mg protein, *P* = 0.001; MIP-2: 5.5 ± 1.9 versus 13.4 ± 1.8 pg/mg protein, *P* = 0.009; IL-6: 8.6 ± 2.5 versus 23.9 ± 2.8 pg/mg protein, *P* < 0.001; Fig. 7a, b, c) and 7 days (MCP-1: 23.7 ± 1.6 versus 41.8 ± 4.9 pg/mg protein, *P* = 0.003; MIP-2: 5.0 ± 2.5 versus 11.8 ± 1.8 pg/mg protein, *P* = 0.003; IL-6: 9.3 ± 1.2 versus 24.3 ± 1.4 pg/mg protein, *P* < 0.001; Fig. 7a, b, c). Similarly, ICH-induced increases in iNOS and COX-2 levels were significantly attenuated by TO901317 treatment. Protein levels of iNOS and COX-2 in TO901317-treated hemorrhagic hemispheres were 35 % (*P* = 0.02) and 42 % (*P* = 0.035) of the vehicle groups, respectively (Fig. 7d, e).

### TO901317 attenuated LPS- and thrombin-induced pro-inflammatory responses in cultured microglia

**Fig. 8:**
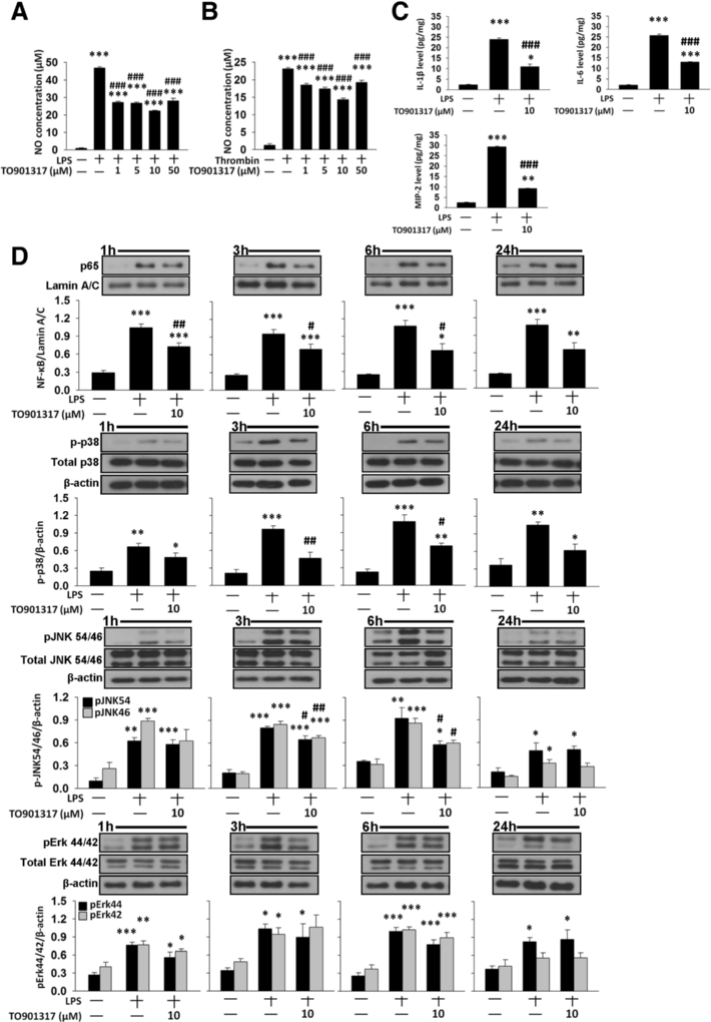
TO901317 inhibited LPS- and thrombin-induced inflammatory responses in cultured microglia. In BV2 microglia, co-treatment of 1, 5, 10, or 50 μM TO901317 with LPS or thrombin for 24 h significantly attenuated **a** LPS- or **b** thrombin-induced release of NO from the supernatant of microglial cultures. **c** Co-treatment of 10 μM TO901317 with LPS for 24 h significantly reduced LPS-induced release of IL-1β, IL-6, and MIP-2 from the supernatant of BV2 microglial cultures. **d** Representative immunoblots and bar graphs show that co-treatment of 10 μM TO901317 with LPS significantly reduced LPS-induced p65 nuclear translocation at 1, 3, and 6 h, p38 and JNK phosphorylation at 3 and 6 h but did not affect Erk phosphorylation in BV2 microglia. Values are presented as mean ± SEM of four independent experiments. ^*^ *P* < 0.05, ^**^ *P* < 0.01, and ^***^ *P* < 0.001 versus normal control; ^#^ *P* < 0.05, ^##^ *P* < 0.01, and ^###^ *P* < 0.001 versus LPS or thrombin stimulation alone (one-way ANOVA)

Our in vivo results demonstrated that TO901317 preserved neuronal function and reduced microglia activation after ICH; thus, we next used mouse BV2 microglial cells to elucidate the underlying molecular mechanisms. We used LPS, a strong immunostimulant, and thrombin, a component of coagulation cascade that is rapidly released following ICH, to activate microglia. The exposure of BV2 microglial cells to LPS for 24 h led to an increase in NO release in the culture supernatant, but co-treatment with 1, 5, 10, or 50 μM TO901317 for 24 h significantly reduced LPS-induced NO production to 58, 57, 48, and 60 % of that observed for the vehicle control group, respectively, and 10 μM TO901317 provided the highest degree of anti-inflammatory action (all *P* < 0.001; Fig. 8a). Therefore, a dosage of 10 μM was employed for subsequent biochemical studies. Similar results were observed after thrombin stimulation (all *P* < 0.001; Fig. 8b). Treatment with 10 μM TO901317 also significantly attenuated LPS-induced production of IL**-**1β (11.0 ± 1.3 versus 24.0 ± 0.7 pg/mg; *P* < 0.001; Fig. 8c), IL-6 (13.0 ± 0.2 versus 25.8 ± 0.6 pg/mg; *P* < 0.001; Fig. 8c), and MIP-2 (9.2 ± 0.1 versus 29.4 ± 0.3 pg/mg; *P* < 0.001; Fig. 8c) as measured in the supernatants of microglial cultures.

We further investigated whether TO901317 influenced the activation of NF-κB, a major transcription factor that regulates pro-inflammatory gene expression and can be activated by mitogen-activated protein kinases (MAPKs), a family of serine/threonine protein kinases that mediate microglial activation [37]. Nuclear NF-κB p65 levels were low in the control group under basal (unstimulated) conditions. Activation of NF-κB, as indicated by nuclear translocation of p65, was observed at 1, 3, 6, and 24 h following LPS stimulation (all *P* < 0.001; Fig. 8d). Co-treatment with 10 μM TO901317 significantly attenuated the LPS-induced increased nuclear levels of NF-κB at 1 h (69 % of vehicle level, *P* = 0.004), 3 h (73 % of vehicle level, *P* = 0.042), and 6 h (61 % of vehicle level, *P* = 0.011) after LPS stimulation (Fig. 8d).

Previous report studies have shown that MAPKs play critical roles in harmful microglial activation during acute brain injury [37]. Therefore, we evaluated the inhibitory effects of TO901317 on LPS-induced activation of MAPKs, including p38, JNK, and Erk. Stimulation of microglia with LPS resulted in rapid activation of p38, JNK, and Erk. Co-treatment with 10 μM TO901317 significantly reduced LPS-induced p38 and JNK phosphorylation at 3 and 6 h (all *P* < 0.05; Fig. 8d). However, there was no difference in the phosphorylation of Erk between TO901317-treated and vehicle-treated groups (all *P* > 0.05; Fig. 8d). Together, these results suggest that TO901317 may inhibit reactive microglial activation and subsequently attenuate the production of pro-inflammatory mediators by suppressing p38 and JNK signaling pathways and NF-κB activation.

### Delayed administration of TO901317 reduced brain damage and neuronal death

**Fig. 9:**
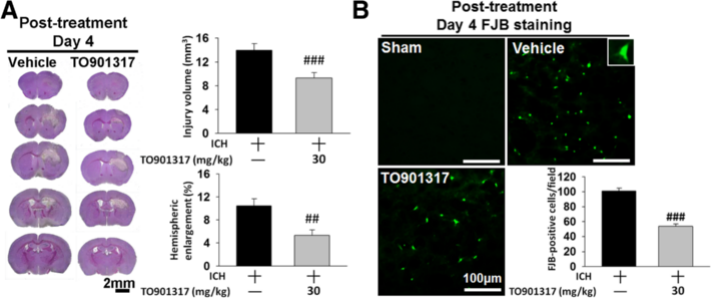
Delayed TO901317 treatment attenuated injury volume, hemispheric enlargement, and neuronal death after ICH. **a** Representative cresyl violet-stained brain sections of a vehicle-treated and a delayed 30 mg/kg TO901317-treated mouse at 4 days post-ICH. Analysis of lesion volumes demonstrates that treatment with 30 mg/kg TO901317 at 3 h post-ICH significantly reduced hemorrhagic injury volume and hemispheric enlargement at 4 days. The scale bar is 2 mm. **b** Representative FJB-stained sections of a sham-injured, a vehicle-treated, and a delayed 30 mg/kg TO901317-treated mouse at 4 days post-ICH. The *inset* is a representative FJB-positive cell at higher magnification. Quantification analysis shows that treatment with 30 mg/kg TO901317 at 3 h post-ICH significantly reduced the number of degenerating neurons at 4 days post-ICH. The scale bar is 100 μm. Values are presented as means ± SEM; ^##^ *P* < 0.01 and ^###^ *P* < 0.001 versus vehicle group (*n* = 7 mice/group, Student’s *t* test)

To determine whether TO901317 still exerts neuroprotective effects when administered at later time points after ICH, TO901317 treatment was delayed by 3 h post-injury. TO901317 treatment at 3 h significantly reduced injury volume (9.3 ± 0.9 mm^3^ versus 14.0 ± 1.1 mm^3^; *P* < 0.001; Fig. 9a) and hemispheric enlargement, an indicator of brain edema (5.3 ± 1.0 % versus 10.5 ± 1.2 %, *P* = 0.0068; Fig. 9a) at 4 days. The number of FJB-positive neurons around the peri-hematoma area was also reduced (53.9 ± 3.1 versus 100.9 ± 4.0 cells/field; *P* < 0.001; Fig. 9b) compared to the vehicle at day 4 post-injury. The neuroprotective effects of TO901317 treatment either immediately after or at 3 h after ICH were similar; injury volume, hemispheric enlargement, and neuronal damage were attenuated by 31, 52, and 51 % when administered immediately post-ICH (Fig. 4) and by 34, 50, and 47 % when treatment started at 3 h post-injury, respectively (Fig. 9).

## Discussion

In this study, we provide the first evidence that activation of LXRs with the synthetic ligand TO901317 improved behavioral outcomes and attenuated brain edema in mice subjected to ICH. Brain tissue damage, neuronal death, and BBB disruption were also reduced following TO901317 treatment. This neuroprotection was observed in conjunction with a reduction of microglial activation, neutrophil infiltration, and expression of inflammatory molecules. Mechanistically, TO901317 attenuated LPS- and thrombin-stimulated NO production in BV2 microglia, which was associated with reduced activation of p38, JNK, MAPK, and NF-κB signaling in LPS-stimulated microglia. Notably, TO901317 was still neuroprotective when using a more clinically relevant treatment time window. Our results suggest that enhancing LXR activation may provide a potential therapy for ICH by modulating the cytotoxic functions of microglia.

We found that LXR-α is selectively upregulated by ICH, although both LXR-α and LXR-β subtypes were detected in the normal brain. Our findings are in agreement with previous reports showing that LXR-α is the form that is predominantly induced in response to a deleterious stimulus such as cerebral ischemia [20] or myocardial ischemia/reperfusion injury [38]. While previous evidence has demonstrated that both LXR-α and LXR-β mediate anti-inflammatory action [39], LXR-α is more responsive in controlling the production of inflammatory molecules compared to LXR-β [40]. On the other hand, whereas LXR-α is expressed predominantly in liver, kidney, intestine, and tissue macrophages, LXR-β is highly expressed in the brain [10, 11]. The importance of LXR-β in brain function is supported by previous studies showing that LXR-β deficiency is associated with central nervous system pathologies and brain development abnormalities [18, 41–43]. For example, LXR-β expression is essential to the formation of the cerebral cortex via its role in guiding the migration of later-born neurons during corticogenesis [41] and is also involved in white matter development and myelination [43]. LXR-β knockout mice exhibit degeneration of motor neurons in the spinal cord and of dopaminergic neurons in the substantia nigra [42]. Furthermore, LXR-β deletion aggravates the loss of dopaminergic neurons in the substantia nigra in an animal model of Parkinson’s disease [18]. Therefore, the specific roles of the individual LXR subtypes in ICH require further clarification.

We showed that TO901317 reduced activation of microglia and ICH-induced upregulation of inflammatory molecules (e.g., MCP-1, MIP-2, IL-6, COX-2, and iNOS) in hemorrhagic brain. In parallel with these in vivo results, TO901317 suppressed LPS- and thrombin-induced production of inflammatory mediators and LPS-induced activation of p38, JNK, and NF-κB pathways in BV2 microglia. Our results suggest a direct effect of LXRs on microglial activation and p38 phosphorylation. Most importantly, we demonstrated that the LXR ligand attenuated inflammation in microglia that were activated by thrombin, which is rapidly produced following ICH and contributes to ICH-induced brain damage [2]. Our findings are in line with previous studies showing that LXR activation suppresses inflammatory responses in animal models of CNS diseases [17–20, 22] and in cultured microglia stimulated with LPS or IL**-**1β [14–16]. LXRs have been reported to deactivate microglia from an amoeboid, active form to a ramified, resting configuration [44]. Following ICH, microglia are activated and then mediate neuroinflammatory responses via producing multiple inflammatory molecules [9], which can activate receptor-dependent apoptotic pathways and contribute to neuronal damage [45]. Experimental evidence has further shown that blocking microglial activation protects against ICH-induced brain damage [46, 47]. In our study, TO901317 reduced neuronal damage at 4 days post-ICH and diminished neurological deficits and decreased brain atrophy at 28 days following ICH. These findings correlated with a reduction of microglia activation in hemorrhagic brain. Thus, it seems very likely that LXR activation may limit brain damage following ICH, in part by blocking microglial activation and attenuating the production of pro-inflammatory mediators.

Our results demonstrated that TO901317 reduced neutrophil infiltration and BBB disruption but did not affect MMP-9 activity. This reduction of infiltrating neutrophils is consistent with previous studies in which TO90317 decreased neutrophil recruitment significantly in damaged spinal cord [19] and lung tissue [48]. The protective effect of TO901317 on neutrophil infiltration may be attributed to a reduction of pro-inflammatory chemokines, which are involved in BBB disruption and the migration of peripheral immune cells into hemorrhagic brain [9]. It is also possible that TO901317 exerts a direct effect on neutrophil responses because a recent study reports that LXR signaling controls peripheral neutrophil homeostasis via regulation of neutrophil clearance and cytokine expression [49]. In contrast with previous reports showing that pharmacological activation of LXRs reduced MMP-9 protein expression following cerebral ischemia [20] and suppressed MMP-9 mRNA and protein expression in macrophages [39], we found that MMP-9 activity was not altered by TO901317 after ICH. One possible explanation for this disparity is that because MMP-9 gene expression is regulated by both NF-κB and activator protein-1 (AP-1) activities [50], antagonism of NF-κB signaling by TO901317 may not be able to suppress AP-1-dependent MMP-9 activity. Another explanation is the time point that was examined. We measured MMP-9 activity at day 4, which is not the peak time point of MMP-9 activation after ICH. Cerebral MMP-9 levels were reported to peak in a bimodal pattern after ICH, with the first peak occurring during days 1–3 and the second peak occurring around day 7 [51]. Indeed, our findings are consistent with previous neuroprotective studies showing that minocycline significantly reduced neuroinflammation and BBB disruption but did not affect MMP-9 levels [27, 52].

Apart from these anti-inflammatory actions, LXRs may provide neuroprotection via other mechanisms. For example, LXR activation by GW3965, a synthetic LXR agonist, suppressed brain beta-amyloid (Aβ) accumulation in a model of mild traumatic brain injury (TBI) in which cerebral inflammation is not significant [53]. This result suggests that the beneficial effects of LXR agonists on mild TBI recovery may be independent from their anti-inflammatory effects. Other studies further show that LXR agonists modulate the deposition and clearance of Aβ metabolism and attenuate Alzheimer’s pathology both in vivo and in vitro [15, 54, 55]*.* In addition to regulating Aβ metabolism, the LXR agonist TO901317 promoted synaptic plasticity and axonal regeneration following experimental cerebral ischemia and increased neurite outgrowth by increasing PI3K signaling in hypoxic cortical neurons [21]. The activation of LXR signaling also protected cultured neurons from oxidative damage-induced toxicity [56]. Furthermore, TO901317 was reported to promote angiogenesis and vascular maturation through eNOS following cerebral ischemia [24]. Whether LXRs exert direct actions on neurons or vessels following ICH warrants further investigation.

## Conclusions

In conclusion, we demonstrated that activation of LXRs by T0901317 reduced functional deficits and brain tissue damage and attenuated neuroinflammation following ICH. T0901317 also reduced activation of p38, JNK, MAPK, and NF-κB signaling in cultured microglia. Considering the extended therapeutic window of T0901317 and its long-lasting effects, our results suggest that pharmacological enhancement of LXRs may have a role as a therapeutic intervention in ICH.

## Additional file

Additional file 1: Figure S1. Effects of 3 different doses of TO901317 on collagenase-induced ICH in mice. (**A**) In all treatment groups, the mNSSs were significantly lower than the vehicle group at 4 and 7 days post-ICH. (**B**) There was no significant difference between the 20 mg/kg TO901317-treated and vehicle-treated groups at all tested time points in the rotarod test. Treatment with 30 mg/kg and 40 mg/kg TO901317 significantly improved rotarod performance compared with the vehicle-treated group at all tested time points following ICH. (**C**) There was no significant difference between the 20 mg/kg TO901317-treated and vehicle-treated groups at all tested time points in the beam walk test. Beam walk latencies were significantly shorter for both the 30 mg/kg and 40 mg/kg groups than the vehicle group at all tested time points following ICH. Values are presented as mean ± SEM; ^#^ *P* < 0.05, ^##^ *P* < 0.01, and ^###^ *P* < 0.001 versus vehicle group (*n* = 12 mice/group, repeated measures two-way ANOVA) (TIFF 1107 kb)

## Abbreviations

ABCA-1: ATP binding cassette transporter-1
ALT: alanine aminotransferase
ANOVA: analysis of variance
BBB: blood-brain barrier
BUN: blood urea nitrogen
COX-2: cyclooxygenase-2
DMEM: Dulbecco’s modified Eagle’s media
DMSO: dimethyl sulfoxide
ELISA: enzyme-linked immunosorbent assay
Erk: extracellular signal-regulated kinases
FJB: Fluoro-Jade B
GFAP: glial fibrillary acidic protein
Iba-1: anti-ionized calcium-binding adaptor molecule 1
IL-1β: interleukin-1β
IL-6: interleukin-6
iNOS: inducible nitric oxide synthase
JNK: Jun amino-terminal kinases
LPS: lipopolysaccharide
LXR: liver X receptor
MAP2: microtubule-associated protein 2
MCP-1: monocyte chemoattractant protein-1
MIP-2: macrophage inflammatory protein
MMP-9: matrix metalloproteinase-9
mNSS: modified neurological severity score
MPO: myeloperoxidase
NF-κB: nuclear factor-kappa B
NO: nitric oxide
PBS: phosphate-buffered saline
SEM: standard error of the mean
