# Supplementary figures and images for: Treatment with TO901317, a synthetic liver X receptor agonist, reduces brain damage and attenuates neuroinflammation in experimental intracerebral hemorrhage
Source: J Neuroinflammation. 2016 Mar 11;13:62. doi: 10.1186/s12974-016-0524-8 (PMC4788882; doi:10.1186/s12974-016-0524-8)

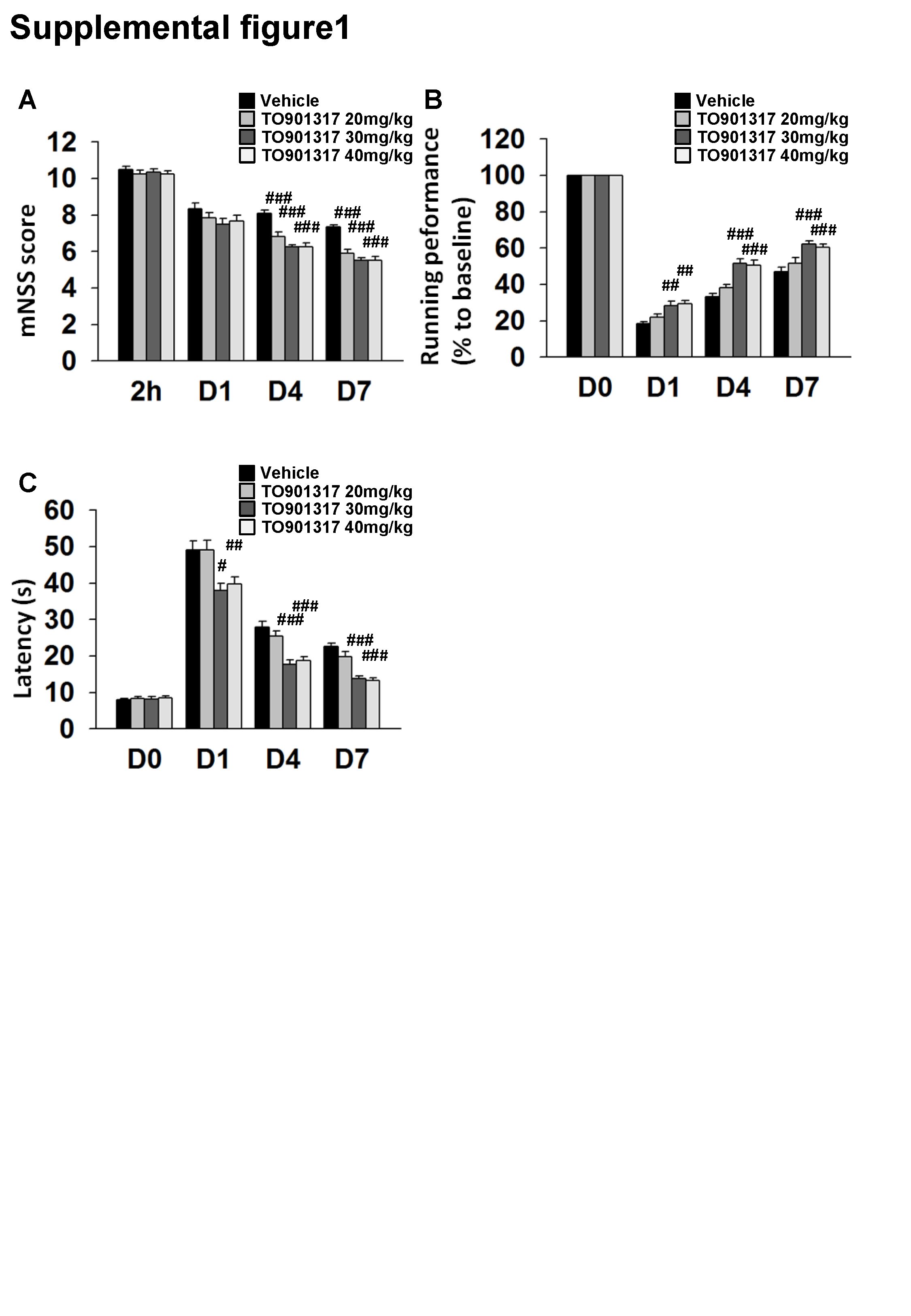

Supplement: Additional file 1: Figure S1. — Effects of 3 different doses of TO901317 on collagenase-induced ICH in mice. (A) In all treatment groups, the mNSSs were significantly lower than the vehicle group at 4 and 7 days post-ICH. (B) There was no significant difference between the 20 mg/kg TO901317-treated and vehicle-treated groups at all tested time points in the rotarod test. Treatment with 30 mg/kg and 40 mg/kg TO901317 significantly improved rotarod performance compared with the vehicle-treated group at all tested time points following ICH. (C) There was no significant difference between the 20 mg/kg TO901317-treated and vehicle-treated groups at all tested time points in the beam walk test. Beam walk latencies were significantly shorter for both the 30 mg/kg and 40 mg/kg groups than the vehicle group at all tested time points following ICH. Values are presented as mean ± SEM; # P < 0.05, ## P < 0.01, and ### P < 0.001 versus vehicle group (n = 12 mice/group, repeated measures two-way ANOVA) (TIFF 1107 kb) [file 12974_2016_524_MOESM1_ESM.tiff]
